# Effects of Platelet-Rich Plasma Injection in Knee Osteoarthritis Stages 1-4: A Retrospective Cohort Study

**DOI:** 10.7759/cureus.111906

**Published:** 2026-07-01

**Authors:** Salah S AlShalahi, Ayyoub Baqer, Jai Shanthini Singaram

**Affiliations:** 1 Physical Medicine and Rehabilitation, Farwaniya Hospital, Al Farwaniyah, KWT; 2 Physical Medicine and Rehabilitation, Jahra Hospital, Al Jahra, KWT

**Keywords:** arab population, kellgren-lawrence grading, knee osteoarthritis, platelet-rich plasma (prp), verbal numerical rating score, womac

## Abstract

Background and aim

Knee osteoarthritis (OA) is a common degenerative joint disorder and a major cause of pain and disability. Platelet-rich plasma (PRP) injections have evolved as an option for the management of knee OA. However, there is a need for studies across different populations and geographic regions. The aim of this study was to assess the outcomes of PRP injections in different stages of knee OA in the Arab population of Kuwait.

Methods

Data were collected retrospectively. Outcome measures were the Verbal Numerical Rating Scale (VNRS) and the reduced Western Ontario and McMaster Universities Osteoarthritis Index (WOMAC). The paired t-test was applied to compare changes in outcomes at three- and six-month follow-up. Effect size was calculated using Cohen’s d standardized mean difference.

Results

In all patients, there was a reduction (p < 0.001) in VNRS at three months (2.87 ± 2.33) and at six months (3.07 ± 2.3) compared with the pretreatment pain score. There was also a reduction (p < 0.001) in WOMAC score at three months (4.8 ± 5.08) and at six months (4.42 ± 5.21). No adverse effects were reported in patients during the study period.

Conclusions

Intra-articular PRP injections may be safe and effective for pain relief and functional improvement for up to six months in all stages of knee OA. To the best of our knowledge, this is the first such study in a large sample of Arab patients.

## Introduction

Osteoarthritis (OA) is a multifactorial disease that causes significant disability and functional limitations [1]. Knee OA is one of the most common conditions encountered in physical medicine and rehabilitation practice. According to the Global Burden of Osteoarthritis Study 2021 [2], there was a 132.2% increase in the number of OA cases in 2020 compared with 1990. The knee was the most commonly affected site. In the Middle East and North Africa (MENA) region, years lived with disability increased by 10% during the same period, and Kuwait ranked among the top three countries with the highest OA burden [3].

The spectrum of treatment options for knee OA ranges from pharmacological, physical, psychosocial, and mind-body approaches [4] to surgery, depending on disease severity. The use of intra-articular biologics in OA has evolved and become increasingly popular worldwide in recent years. Platelet-rich plasma (PRP) injections have been found to provide longer-lasting benefits [5] than other injectable therapies. Platelets are a rich source of growth factors, including fibroblast growth factors, insulin-like growth factor-1, transforming growth factor-β1, vascular endothelial growth factor, and bone morphogenetic proteins, which enhance tissue regeneration and healing [6].

The National Institute for Health and Care Excellence recommendation for the use of PRP injections in knee OA [7] states that it is a safe procedure and encourages further research. Currently, the clinical uncertainty regarding the use of PRP is less than it was a decade ago. Although concerns continue to be raised about its efficacy [8] and the supporting evidence base, an increasing number of randomized controlled trials [9], systematic reviews, and meta-analyses [10,11] have demonstrated that PRP has an important role in the management of knee OA. These studies also highlight the need for larger studies [12] across diverse populations.

A literature search of PubMed for studies evaluating intra-articular PRP injections for knee OA in the MENA region revealed a paucity of clinical studies. This study aimed to assess the clinical and functional outcomes of intra-articular PRP injections across different stages of knee OA. To the best of our knowledge, this is the first large multicenter study of its kind in an Arab population.

## Materials and methods

Study design and setting

This retrospective pre-post observational study was conducted in the physical medicine and rehabilitation outpatient clinics at Farwaniya Hospital and Jahra Hospital, Kuwait, between August 2023 and July 2024. The study was reported according to the STrengthening the Reporting of OBservational studies in Epidemiology (STROBE) guidelines [13]. Written informed consent was obtained from all patients before treatment.

Subjects

We searched the records of patients who underwent PRP injection for a diagnosis of knee OA. To be eligible for PRP injection, adults diagnosed with knee OA had to have chronic knee pain that persisted despite usual treatment, including analgesics, cartilage supplements, hyaluronic acid injections, and physical therapy. Participants were included regardless of sex or OA severity. Patients with bilateral knee OA were included. Participants with platelet dysfunction disorders, severe thrombocytopenia, or acute systemic illness were excluded from receiving PRP injections.

PRP preparation and characteristics

Using an FDA-approved device, PRP was prepared according to the manufacturer’s instructions using single-use patented kits. The kit consisted of a 10-mL glass vacuum tube certified to maintain standardized results through an easily reproducible technology, resulting in autologous leukocyte-poor PRP with a platelet concentration factor of 1.6×. The same type of kit and technique were used for every patient [14]. Blood was collected from each patient using a sterile technique and centrifuged at 1500 relative centrifugal force for five minutes. After centrifugation, approximately 5-6 mL of PRP was withdrawn from the kit for injection.

Procedure for PRP injection

All patients who underwent PRP therapy were instructed to stop using nonsteroidal anti-inflammatory drugs (NSAIDs) three days before the injection. The intra-articular injection was performed using the standard technique described in the literature [15]. Universal precautions were observed during the procedure. Patients were given post-injection instructions, including avoiding NSAIDs for two weeks and performing home exercises such as isometric quadriceps and joint range-of-motion exercises. All patients were scheduled for regular follow-up appointments. Detailed records were maintained for each patient.

Assessment and outcome measurements

The following assessments and measurements were collected from patients’ records: (A) demographics and OA characteristics: age, sex, BMI, time since OA diagnosis, and Kellgren-Lawrence (KL) grading [16] of OA severity; (B) outcome measurements: outcome measurements assessed before PRP intervention and at three- and six-month follow-up, including (i) the Verbal Numerical Rating Scale (VNRS) [17], in which patients were asked to rate their pain on a scale of 0 to 10, where 0 indicates no pain and 10 indicates the worst imaginable pain; and (ii) the reduced Western Ontario and McMaster Universities Osteoarthritis Index (WOMAC) [18,19], a self-administered patient-reported outcome measure consisting of seven questions assessing function. Each question is scored from 0 to 4, and the total score ranges from 0 to 28, with lower scores indicating better function; and (C) adverse events or side effects.

Statistical analysis

Statistical analysis was performed using IBM SPSS Statistics for Windows, version 21.0 (released 2012; IBM Corp., Armonk, NY, USA). Patient de-identification was performed by removing personal identifiers from the collected data. Parametric tests were used based on the results of the Shapiro-Wilk normality test, which confirmed that the data were normally distributed. Descriptive statistics, including mean, SD, frequency, and percentage, were calculated. The paired t-test was applied to compare changes in outcomes at three- and six-month follow-up. Effect size was calculated using Cohen’s d standardized mean difference. Cohen’s d was calculated as the difference between the pre- and post-procedure means divided by the pooled SD.

## Results

A total of 119 knees from 91 patients were identified in our search. Of these, 114 (95.8%) knees from 86 (94.5%) patients were included in the analysis. Five patients were excluded from the study. Of these five patients, two had undergone a surgical procedure, and three had sustained new trauma to the treated knee. Twenty-three (26.74%) patients were male, and 63 (73.26%) were female. The mean age was 59.97 ± 9.98 years, the mean BMI was 33.91 ± 6.06 kg/m², and the mean duration of symptoms was 4.39 ± 3.64 years (Table 1).

**Table 1:** Baseline demographics and clinical characteristics in each grade of knee OA KL, Kellgren-Lawrence; OA, osteoarthritis

Variable	KL stage 1 (n = 12)	KL stage 2 (n = 37)	KL stage 3 (n = 23)	KL stage 4 (n = 42)	Total (N = 114)
Number of knees (%)	12 (10.5)	37 (32.5)	23 (20.2)	42 (36.8)	114 (100)
Age, mean ± SD (years)	53.08 ± 7.83	56.27 ± 6.76	58.26 ± 9.99	66.14 ± 9.87	59.97 ± 9.98
Duration of illness, mean ± SD (years)	2.08 ± 2.64	3.32 ± 3.05	4.47 ± 4.29	5.95 ± 3.40	4.39 ± 3.64
BMI, mean ± SD (kg/m²)	32.14 ± 4.59	32.24 ± 4.78	35.26 ± 6.18	35.16 ± 7.00	33.91 ± 6.06

The mean WOMAC and VNRS scores at baseline were 18.27 ± 7.83 and 7.22 ± 2.47, respectively. The WOMAC and pain scores improved at three- and six-month follow-up (p < 0.001). The Cohen’s d effect size for WOMAC was 2.12 at three months and 2.07 at six months of follow-up, while for VNRS it was 1.86 at three months and 1.76 at six months of follow-up. The improvements were observed across all stages of knee OA. The improvement in WOMAC was greater in more severe stages; the effect size in KL stages 3 and 4 was larger than in KL stages 1 and 2. Conversely, the improvement in VNRS was greater in milder stages; the effect size in KL stages 1 and 2 was larger than in KL stages 3 and 4. Changes in pain and WOMAC scores at baseline and follow-up are shown in Table 2.

**Table 2:** Distribution of WOMAC and pain scores at baseline and during patient follow-up at three and six months Values are presented as mean ± SD. * The paired t-test was used to compare WOMAC (reduced) and pain (VNRS) scores at baseline with three and six months. ** The nonparametric test (related-samples Wilcoxon signed-rank test) was used to compare WOMAC and pain (VNRS) scores at baseline with three and six months. Change scores represent reduction from baseline (negative values indicate improvement). Effect sizes are reported as Cohen’s d (mean change divided by the SD of the change), appropriate for within-group pre-post designs. Magnitude interpretation follows Cohen’s criteria: 0.2 = small, 0.5 = medium, 0.8 = large. KL, Kellgren-Lawrence; VNRS, Verbal Numerical Rating Scale; WOMAC, Western Ontario and McMaster Universities Arthritis Index

Variable	KL stage 1 (n = 12)	p-value	KL stage 2 (n = 37)	p-value	KL stage 3 (n = 23)	p-value	KL stage 4 (n = 42)	p-value	Total (n = 114)	p-value
WOMAC										
Baseline	13.92 ± 9.25	-	17.0 ± 8.54	-	19.13 ± 7.26	-	20.17 ± 6.51	-	18.27 ± 7.83	-
Three months	1.42 ± 1.25	<0.001* / <0.003**	3.22 ± 4.50	<0.001* / <0.001**	4.48 ± 3.50	<0.001* / <0.001**	7.33 ± 5.83	<0.001* / <0.001**	4.80 ± 5.08	<0.001* / <0.001**
Change	-12.5 ± 9.2		-13.8 ± 7.8		-14.7 ± 6.6		-12.8 ± 6.0		-13.47 ± 7.07	
Effect size (d)	1.63	-	1.94	-	2.33	-	2.17	-	2.12	-
Six months	1.42 ± 1.25	<0.001* / <0.002**	2.49 ± 4.14	<0.001* / <0.001**	4.48 ± 3.50	<0.001* / <0.001**	6.95 ± 6.34	<0.001* / <0.001**	4.42 ± 5.21	<0.001* / <0.001**
Change	-12.5 ± 9.2		-14.5 ± 8.2		-14.7 ± 6.7		-13.2 ± 6.4		-13.85 ± 7.29	
Effect size (d)	1.63	-	2.1	-	2.33	-	2.11	-	2.07	-
VNRS										
Baseline	6.83 ± 3.19	-	7.22 ± 3.19	-	7.04 ± 2.40	-	7.43 ± 2.15	-	7.22 ± 2.47	-
Three months	1.33 ± 0.78	<0.001* / <0.005**	2.27 ± 2.26	<0.001* / <0.001**	2.83 ± 2.17	<0.001* / <0.001**	3.86 ± 2.39	<0.001* / <0.001**	2.87 ± 2.33	<0.001* / <0.001**
Change	-5.5 ± 3.2		-4.9 ± 3.1		-4.2 ± 2.9		-3.6 ± 2.8		-4.35 ± 2.99	
Effect size (d)	2.19	-	1.74	-	1.85	-	1.58	-	1.86	-
Six months	1.33 ± 0.98	<0.001* / <0.005**	2.41 ± 2.25	<0.001* / <0.001**	3.30 ± 2.18	<0.001* / <0.001**	4.02 ± 2.25	<0.001* / <0.001**	3.07 ± 2.30	<0.001* / <0.001**
Change	-5.5 ± 3.1		-4.8 ± 3.3		-3.7 ± 3.0		-3.4 ± 2.8		-4.15 ± 3.11	
Effect size (d)	2.08	-	1.63	-	1.59	-	1.52	-	1.76	-

**Figure 1:**
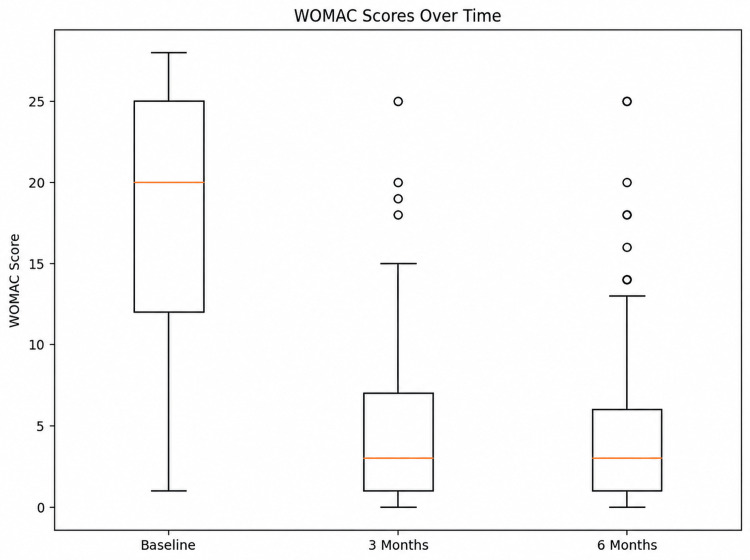
Box plots depicting changes in WOMAC scores at baseline, three months, and six months after treatment The central line represents the median score; boxes represent the IQR; whiskers extend to 1.5×IQR; individual points indicate outliers. WOMAC, Western Ontario and McMaster Universities Arthritis Index

**Figure 2:**
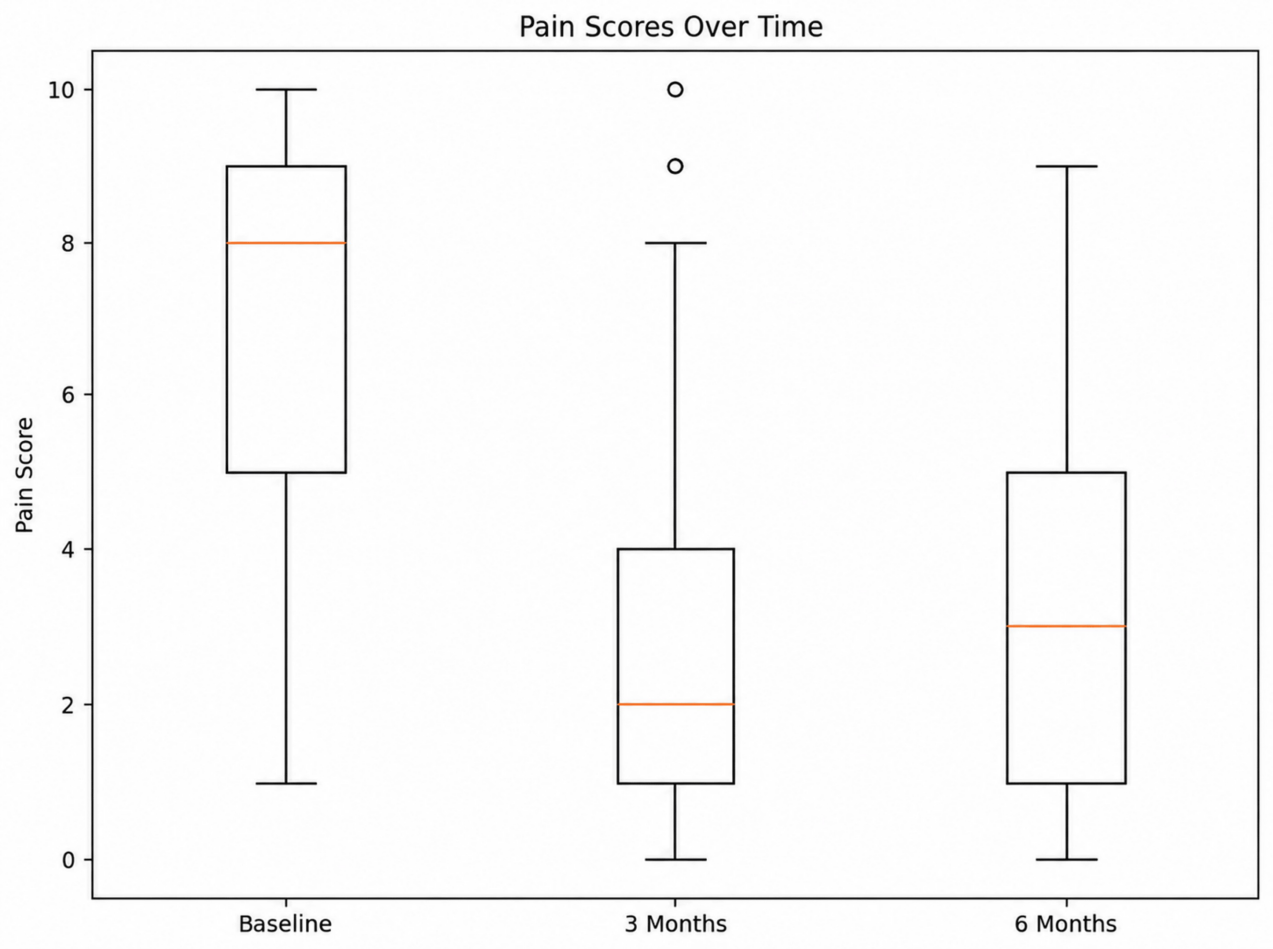
Box plots depicting changes in pain scores at baseline, three months, and six months Medians are shown as central lines, IQRs are represented by boxes, whiskers indicate 1.5×IQR, and outliers are shown as individual points.

A large proportion of patients experienced improvement at the three-month follow-up, and most continued to benefit from the treatment at six months (Figures 1, 2).

According to WOMAC scores, patients’ functioning was more stable over time than pain levels, suggesting that functional improvements may be maintained even when pain relief varies. The presence of a small number of outliers reflects variability among patients with OA and the need for individualized monitoring. No adverse events or side effects were reported.

## Discussion

Efficacy of PRP injection in knee OA

Through this study, we performed an overall assessment of the efficacy of intra-articular PRP injections in routine care. PRP injections provided clinical benefits in patients across all degrees of severity and persisted for up to six months. The results of our study are in line with those of other studies. The efficacy of PRP in relieving pain and improving function, stiffness, and quality of life in patients with knee OA for up to 12 months is supported by Mende et al. [20].

PRP effects in various stages of knee OA

In our study, the mean WOMAC and pain scores before and after treatment were progressively higher across OA severity grades, indicating a lower response in more severe OA. In addition, more severe grades showed a smaller change in both WOMAC and pain scores. As the natural course of disease progresses, the efficacy of PRP appears to decrease. In a similar study of 89 patients by Annaniemi et al. [21], all patients with knee OA stages 1-3 treated with three PRP injections showed a decrease in WOMAC and VAS (Visual Analogue Scale) scores; however, patients with mild knee OA benefited more than those with more severe disease. KL stage 1 patients had a slight advantage over KL stages 2 and 3. The authors concluded that PRP injections have a diminishing effect as OA progresses; however, meaningful effects are observed across KL stages 1-3, with the greatest benefit in KL stage 1 knee OA.

There are fewer studies on the effect of PRP injections in stage 4 OA compared with other stages. Opinions vary regarding the role of PRP in stage 4 OA. Given the extent of damage in severe knee OA, it is reasonable to question the indication for regenerative injections in severely affected knees. In a systematic review, Anzillotti et al. [22] analyzed the rationale for injectable orthobiologic treatments, including PRP, in patients with severe knee OA. They reported that the lack of uniform beneficial effects does not support routine use of orthobiologics in severe stage 4 OA as standard care. In our study, 36.84% of treated knees were stage 4 OA. Although changes in both pain and functional scores were observed at three and six months in these patients, the overall improvement was smaller compared with mild and moderate OA.

There is evidence suggesting that PRP may serve as a bridge therapy in patients awaiting knee replacement surgery. In a prospective randomized double-blinded clinical trial of 65 patients with advanced knee OA, Joshi Jubert et al. [23] observed that a single intra-articular PRP injection was effective in relieving pain and improving activities of daily living and quality of life at six-month follow-up. The response to PRP in stage 4 OA may also depend on other factors such as age and knee deformity. Sánchez et al. [24] performed a five-year survival analysis of 481 patients with stage 3 and 4 knee OA who received PRP injections and reported that 85.6% did not undergo total knee arthroplasty (TKA). In stage 4 knee OA, only patients younger than 65 years avoided TKA over the five-year period. Their data suggested that PRP may delay TKA in selected patients. In the same study, among 186 patients who eventually underwent TKA after PRP treatment, arthroplasty was delayed by more than 1.5 years in 74.1% of cases, with a median delay of 5.3 years, supporting the use of PRP in selected stage 4 OA patients.

In our study, we excluded one patient who underwent knee replacement from the analysis. The mean age of patients with stage 4 OA was 66.14 ± 9.87 years. In a retrospective cohort study, Saita et al. [25] observed that knee deformity was a significant predictor of reduced improvement. Severe varus deformity (>190°) diminished the effectiveness of PRP therapy. Even in severe OA, effectiveness was not reduced when the femorotibial angle was <180°. In our study, the femorotibial angle was not measured. However, in our practice, patients with advanced OA and clinical varus deformity were advised to consider alternative treatments and were therefore naturally excluded from this study.

Arab population

The growing awareness of PRP among patients places an ethical responsibility on providers to address research needs in diverse populations [26]. Studies on racial differences have observed no significant differences in the utilization of intra-articular steroids and hyaluronic acid injections among Caucasians; however, data on differential treatment response remain limited [27]. There is a paucity of demographic data in PRP studies [28]. A study in Saudi Arabia involving 225 knees [29] showed that PRP improved pain relief and knee flexion in patients with stage 2-4 OA for up to nine months. That study used the Numerical Rating Scale and goniometric measurement of knee flexion. In the current study of 114 knees, in which 98.25% of patients were of Arab descent, VNRS and WOMAC scores were used to assess pain and functional improvement. We also observed that patients with high BMI responded favorably. In our cohort, BMI was >35 in 57.01% of patients.

Strength of the study and clinical implications

The main strength of this study is the large sample size of a native population treated in two major regional general hospitals. In addition, the same type of PRP kit was used for all patients. One of the common dilemmas in treating knee OA is whether to use intra-articular PRP injections. Our study, conducted on a large sample of Arab patients across different stages of knee OA, may help support more rational clinical decision-making.

Limitations

The main limitation of this study is its observational design, which increases the risk of bias and confounding. However, the adequate sample size, clear definition and classification of knee OA, and standardized PRP preparation technique may partially mitigate this risk. The statistical analysis could be further improved to account for confounding factors. Although this study was conducted in a specific geographic population and stratified by OA stage through subgroup analysis, potential confounders such as degenerative conditions of the spine and hip may have influenced pain and functional scores. These could have been better addressed using more advanced analytical methods. The use of a mixed-effects model in this multicenter study, in which 35.55% of patients received bilateral knee injections, could have improved the robustness of the analysis.

## Conclusions

Intra-articular PRP injections demonstrated favorable short-term observational outcomes in a selected cohort of patients with various stages of knee OA. Further controlled studies are warranted to confirm their long-term efficacy and safety. Larger sample sizes and standardized protocols could strengthen the evidence base for their use in different clinical settings. The availability of other cost-effective treatments with substantial outcomes may influence the design of future large-scale studies.
